# The Paddington Fund for the Unemployed

**Published:** 1888-07-14

**Authors:** 


### THE PADDINGTON FUND FOR THE UNEMPLOYED.

*See* THE HOSPITAL, *vol. iii., No.* 64, *Dec.* 17, 1887, "*London's Great Problem Solved.*"

LAST winter there was made in Paddington a more systematic attempt than in any other of the districts of the metropolis to provide work for the unemployed poor, as a substitute for the promiscuous alms-giving which had been found too often to aggravate the distress it was meant to cure. The method employed was on the whole so very successful in attaining its object that a brief account of its operations may be worth narrating as a guide to other districts and other towns that may adopt a similar plan.

The necessary funds having been supplied by a comparatively small number of residents within the electoral district of Paddington, it was advisable that the bestowal of it should be confined to the poor of the same district, a limitation which, however harsh it seems, is inevitable, as otherwise the poor of every district in London would have flocked in, and the fund distributed among so large a number would have done no appreciable good. In dealing with a distress which is likely to last for several months, it is well to go on the principle of keeping one man for a week rather than seven for a day. Beyond this limitation the only primary qualification was residence in the district for at least three months. The fund was meant for residents, not for tramps and casual comers, and to prevent members of these classes taking up their abode in Paddington just in time to secure this qualification, it is proposed that in future winters the residence qualification shall be one year and three months.

It is only, however, when an applicant has proved so far suitable that the real investigation of the case begins.

No application was received except through the recommendation of some responsible society or known householder of the district, who was expected to fill up a form which gave when completed a brief history of the individual recommended —his name, age, and address, his regular occupation, and the weekly wages he earned by it, the length of time he had been out of work, the assistance—if any—he had received from other sources, his weekly rent, and how much it was in arrears, the number of children he had, and all particulars of his family; whether he was a member of any benefit society, whether his tools and other articles were in pawn; finally, the name and address of his last employer, and a reference to some clergyman who knew him.

These questions being answered in an apparently satisfactory form, two more forms were sent out to be filled, one to the clergyman referred to, asking if the applicant was being assisted out of any fund at his command, and if so, to what extent; the other to the person stated to be the last employer, asking if the statements made by the applicant were true. Of course if he had been guilty of any false statement in this matter it cast a doubt over his case. It sometimes transpired, however, that the employer knew the man under some nickname or appellation other than that he gave the committee; this would give rise to a new series of inquiries, but did not inevitably prejudice the case. The employer was asked also why the applicant had left his service—was it slackness of trade, misconduct, or any other cause.

If the applicant passed satisfactorily through all these investigations the words "passed for employment" were written against his case-form, and work was found for him as soon and as suitably as possible. The vestry provided work for some, the largest number employed by them in a week being sixty-three, while the making of a recreation-ground employed more, sometimes as many as 137 a week. For this labour less than the ordinary rate of pay was given, in order that there might be no temptation to be provided with work in this way rather than in the open market. In some cases the applicant's story ran that Mr. So-and-So would give him a job if he had tools, or it might be, as in the case of an old waiter, suitable clothes. In these cases inquiries were made of the employer mentioned, and if he confirmed the applicant's statement the tools or clothes were taken out of pawn, and the man provided for in that way.

On being "passed for employment," and employed at the recreation-ground, an applicant was kept on for a month, if he did not meanwhile obtain employment elsewhere. At the end of a month the cases were reconsidered. If there were other claimants for whom work had to be found a number of unmarried men were dismissed; married men—those who had a family to keep together, and to whom, therefore, going into the workhouse (if no alternative was provided) was a more serious and fatal step than in the case of the unmarried were kept on.

For women a workroom was provided on exceptionally generous principles. The women taken on there—whose case-form was similar to that of the men, but rather less elaborate—were paid one shilling for four hours' work, from one o'clock to five—hours when they could best be spared from their domestic duties. They were not employed on work for sale, but in mending their own and their children's clothes. It will surprise no one who has gone much among the wives of our working men to learn that when they went to the work-room many of them knew nothing of even the rudiments of needlework, and had to be taught how to patch and darn their garments.

But, it will be said, while all these inquiries—useful and necessary, no doubt—were being made, the applicants and their families might be starving. It was, however, in the power of the committee to give relief in money at their discretion if the distress was proved to be immediate and serious. The whole ground for the benefit of whose poor the money was collected was divided into small districts, each of which had a voluntary almoner. On him devolved the duty of making personal inquiry into urgent cases, and giving money or food where it was evidently required at once. This pecuniary relief, however, was limited to 3*s*. a-week for each adult and 1*s*. a week for each child. No family ever received more than 10*s*. a-week in all; and the relief was never granted for more than two weeks without reconsideration of the case.

This, in brief, was the method by which the fund was distributed. It is based primarily on thorough personal investigation and a strict determination to exclude from the benefits of the fund as far as possible the idle and ill-conducted who are out of employment through their own fault; and, secondly, to make all relief granted take the form of work rather than of gifts. It required the earnest and conscientious co-operation of a considerable band of voluntary labourers, of whom was demanded sufficient justice to sift a case to the bottom without carelessness or prejudice, and sufficient determination to refuse help when they saw it was not deserved.

The committee, reviewing their labours, consider that this has been done, and that the moral results of the distribution of the fund have been satisfactory. They have helped the poor without pauperising them; have enabled families that would otherwise have been forced to go to the workhouse—with the loss of family feeling and personal responsibility that step so often entails—to tide over the winter and be ready and fit for their ordinary work when it could be obtained. Finally, it is not unlikely that the hard but honest work they offered has helped to keep the young from drifting into crime.

The statistical results are these: 874 persons applied for work, of whom 643 obtained it through the committee; 57 found or were assisted to private work; 135 were unprovided for, most of these being, for some reason or other, ineligible. The applicants represented 56 different occupations, exclusive of the 284 women who sought for help. The total sum expended in providing work, redeeming tools, and reinstating members of benefit societies, and giving relief in money, coals, food, &c., was £1,838 12*s*. 6*d*.; while the total expenses connected with the distribution of this sum amounted to £120 15*s*. 2*d*. This result could not have been obtained so cheaply if great part of the work had not been done voluntarily, and the result seems to justify the growing conviction that any good done to poor of a district in times of distress will depend chiefly on the individual efforts as well as the ndividual offerings of their fellow-residents.

